# Secondary syphilis mimicking marginal zone B-cell lymphoma in a 70-year-old man

**DOI:** 10.1016/j.jdcr.2025.08.006

**Published:** 2025-08-25

**Authors:** Hannah Gier, Shannon Trotter

**Affiliations:** a Conway Medical Center, Conway, South Carolina; b Department of Dermatology, OhioHealth, Columbus, Ohio

**Keywords:** clonal B cells, differential diagnosis, histopathology, immunohistochemistry, lymphoproliferative disorders, marginal B-cell lymphoma, secondary syphilis, spirochete infection, syphilis mimics

## Case description

**Fig 1:**
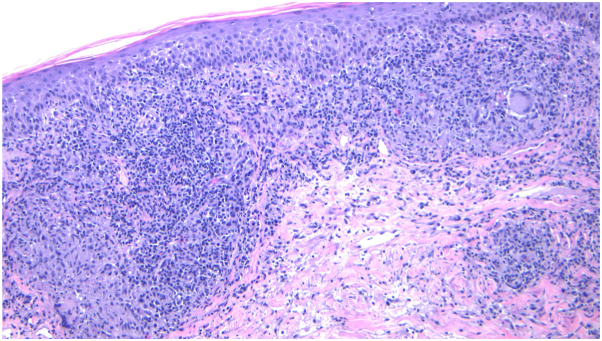
10**×** magnification: dense superficial and deep perivascular and lichenoid lymphocytic infiltrate.

**Fig 2:**
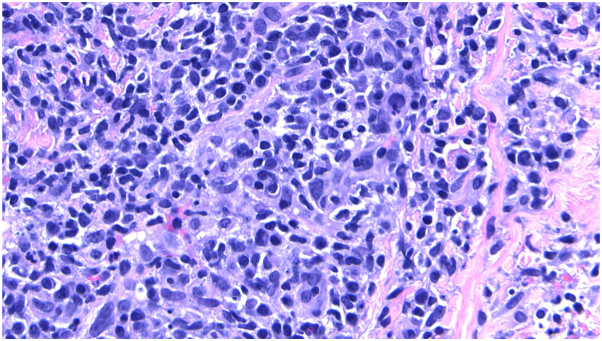
40**×** magnification: prominent perivascular lymphocytic infiltrate with numerous plasma cells.

**Fig 3:**
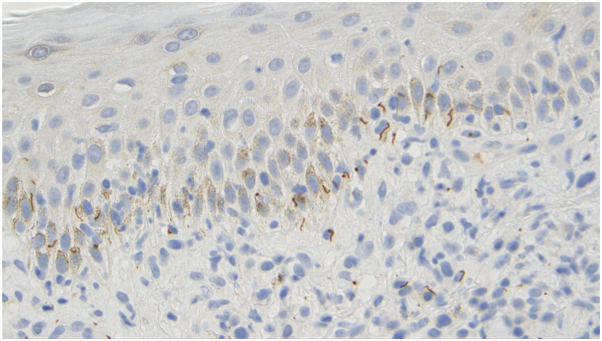
Immunoperoxidase stain highlighting treponemal organisms within the tissue.

A 70-year-old man presents with a 4-year history of a pruritic eruption involving the trunk, head, neck, and extremities. He reports intermittent fever, chills, and fatigue. Positron emission tomography/computed tomography imaging demonstrates diffuse hypermetabolic cervical, axillary, and inguinal lymphadenopathy, raising concern for lymphoma. A bone marrow biopsy reveals clonal B cells. Skin examination shows erythematous papules and nodules with overlying scale. A skin biopsy demonstrates compact orthokeratosis, angiocentric and lichenoid lymphocytic inflammation, and granulomas with abundant plasma cells. Immunohistochemical staining for *Treponema pallidum* is positive, and the rapid plasma reagin (RPR) titer is 1:2048. Flow cytometry identifies low-level monoclonal B cells without atypia (Fig 1, Fig 2, Fig 3).

**Fig 4:**
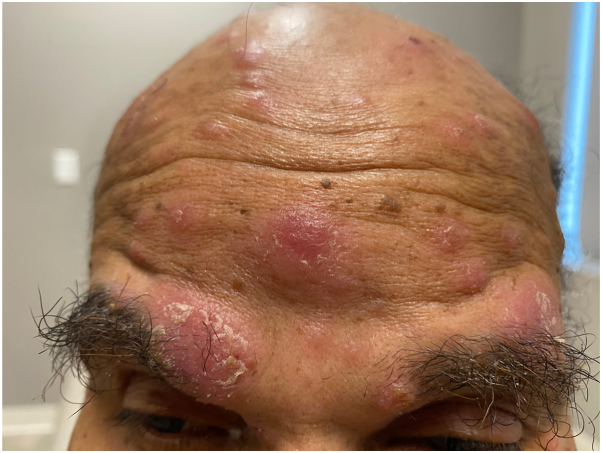
Erythematous plaques and nodules with overlying scale involving the forehead, glabella, eyebrows and scalp at initial presentation.

**Fig 5:**
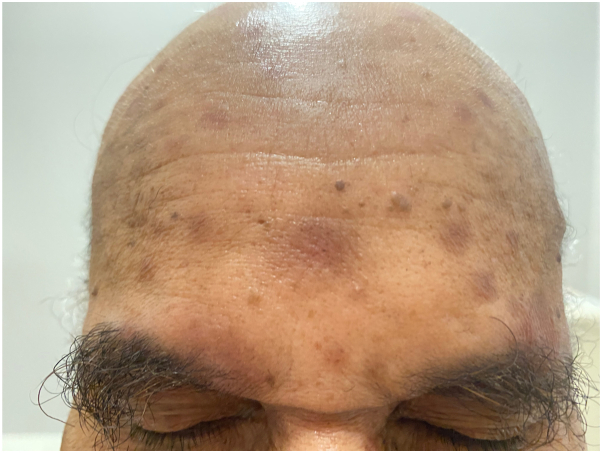
Marked clinical improvement after a 28-day course of doxycycline 100 mg twice daily. Residual post-inflammatory hyperpigmentation on the forehead, glabella, eyebrows and scalp.

The patient is diagnosed with secondary syphilis. Due to a penicillin allergy, he is treated with doxycycline 100 mg twice daily for 28 days, resulting in marked clinical improvement. His papulonodular eruption significantly regresses, with decreased erythema and residual postinflammatory hyperpigmentation. The patient’s systemic symptoms also resolve, and follow-up RPR titers decrease to 1:4 (Figs 4 and 5).

**Question 1: Which of the following best explains the presence of clonal B cells in this patient with a reactive lymphoplasmacytic infiltrate and positive treponemal stain?**

**A.** Early marginal zone lymphoma with secondary infection
**B.** Nodal involvement by cutaneous pseudolymphoma
**C.** Sampling error from skin adjacent to a neoplastic focus
**D.** Clonal B-cell expansion secondary to infection
**E.** Technical artifact during flow cytometry

**Correct answer: D. Clonal B-cell expansion secondary to infection.**

## Discussion

This case highlights a rare example of secondary syphilis mimicking marginal zone B-cell lymphoma. Though the presence of clonal B cells often suggests a lymphoproliferative disorder, reactive conditions like syphilis can also induce B-cell clonality.^1^^,^^2^ Clonal redemption is a process in which memory B-cell clones resurge in response to an antigenic stimulation caused by a pathogen, such as *T pallidum*, without malignant transformation. This phenomenon can complicate diagnosis and lead to misdiagnosis as lymphoma if proper clinicopathologic correlation is not performed.

Histologically, marginal zone B-cell lymphoma and secondary syphilis may share features, including plasma cell-rich infiltrates and dermal lymphoid aggregates.^1^, ^2^, ^3^, ^4^ However, the presence of spirochetes on treponemal immunostaining, positive RPR and treponemal antibody serologies, and clinical response to antibiotic therapy all support an infectious rather than neoplastic etiology. Additional histologic features that favor syphilis include a lichenoid band of inflammation, elongated rete ridges, and endothelial swelling.^2^^,^^3^

While secondary syphilis is a known mimic, this case shows a lesser-reported manifestation resembling cutaneous B-cell lymphoma both clinically and histologically, with reactive B-cell clonality further complicating diagnosis in an atypical demographic. It emphasizes the need to integrate histopathologic, immunohistochemical, and serologic findings, as clonal redemption remains an underrecognized mechanism that can mislead clinicians toward malignancy.

## Conflicts of interest

None disclosed.
